# Increasing the Effectiveness of Pharmacotherapy in Psychiatry by Using a Pharmacological Interaction Database

**DOI:** 10.3390/jcm10102185

**Published:** 2021-05-18

**Authors:** Michal Ordak, Tadeusz Nasierowski, Elzbieta Muszynska, Magdalena Bujalska-Zadrozny

**Affiliations:** 1 Department of Pharmacodynamics, Centre for Preclinical Research and Technology (CePT), Medical University of Warsaw, 02-091 Warsaw, Poland; mbujalska@gmail.com; 2 Department of Psychiatry, Medical University of Warsaw, 02-091 Warsaw, Poland; tadeusz@wum.edu.pl; 3 Department of Medical Biology, Medical University of Bialystok, 15-089 Bialystok, Poland; ela3@onet.eu

**Keywords:** pharmacological interaction database, new psychoactive substances

## Abstract

Recent studies have shown that the knowledge of pharmacological interaction databases in global psychiatry is negligible. The frequency of hospitalizations in the case of patients taking new psychoactive substances along with other drugs continues to increase, very often resulting in the need for polypharmacotherapy. The aim of our research was to make members of the worldwide psychiatric community aware of the need to use a pharmacological interaction database in their daily work. The study involved 2146 psychiatrists from around the world. Participants were primarily contacted through the LinkedIn Recruiter website. The surveyed psychiatrists answered 5 questions concerning case reports of patients taking new psychoactive substances along with other drugs. The questions were answered twice, i.e., before and after using the Medscape drug interaction database. The mean percentage of correct answers given by the group of psychiatrists who were studied separately in six individual continents turned out to be statistically significantly higher after using the pharmacological interaction database (*p* < 0.001). This also applies to providing correct answers separately, i.e., to each of the five questions asked concerning individual case reports (*p* < 0.001). Before using the drug interaction database, only 14.1% of psychiatrists stated that they knew and used this type of database (*p* < 0.001). In the second stage of the study, a statistically significant majority of subjects stated that they were interested in using the pharmacological interaction database from that moment on (*p* < 0.001) and expressed the opinion that it could be effective in everyday work (*p* < 0.001). Using a pharmacological interaction database in psychiatry can contribute to the effectiveness of pharmacotherapy.

## 1. Introduction

Mental disorders are a very serious and increasing problem worldwide. According to the World Health Organisation (WHO), the results of the Global Burden of Diseases study show the importance of mental health in the area of public health. Based on WHO forecasts, depression will be at the top of the list of the most common diseases in the world in 2030 [1]. A recent report shows that as many as 25 million Europeans (5.4% of the population) suffered from anxiety, 21 million (4.5%) suffered from depression or depressive conditions, 11 million (2.4%) suffered from alcohol and/or drug addiction, and around 1.5 million (0.3%) people suffered from schizophrenia spectrum disorders [2]. A report prepared by a team of experts from all over the world shows that the mental health crisis is increasing significantly and health systems are not responding to it effectively enough. It has been estimated that by 2030, the cost of this crisis will be approximately $16 trillion [3].

In recent years, there has been, among other things, a sharp increase in the frequency of the hospitalization of patients receiving new psychoactive substances [4,5,6]. Most patients who abuse different types of new psychoactive substances combine them with other addictive substances. There is an increasing number of hospitalisations of patients taking different types of new psychoactive substances continuously, and thus the need to treat these patients with multiple drugs at the same time increases the risk of pharmacological interactions not only between the administered drugs but also between uncontrolled addictive substances. This often makes it significantly more difficult to achieve a therapeutic effect and is one of the factors that increase the risk of re-hospitalisation as well as increases the costs incurred by a country [7]. For example, in the group of patients abusing mephedrone, it has been found that the frequency of hospitalisations increases in correlation with the number of psychotropic drugs taken simultaneously [8].

Adverse reactions associated with different types of drug interactions result in numerous health losses and contribute to an increased frequency of patient hospitalisations and deaths [9,10,11]. For example, in the United States, the cost of treatment due to medical errors and side effects in the elderly is estimated at over USD 200 billion annually [12]. The same applies to European countries [13,14]. The number of patients who are under the influence of multiple substances during admission to hospital in different countries is estimated at 20–60% and is also a reason for rehospitalisation [15,16,17,18]. Reducing the number of avoidable readmissions is essential to current healthcare policies. Knowledge of the consequences of drug interactions is crucial for planning the pharmacotherapy process. Noticing the actual scale of this phenomenon in the group of people taking new psychoactive substances emphasises the importance of joint efforts that should be taken by the medical community to improve the quality of pharmacotherapy [19].

Nowadays, there are more and more pharmacological databases available where you can check whether or not there is a dangerous drug interaction. Unfortunately, as it turns out, the frequency of use of these databases in psychiatry is almost negligible. In a recently published article in Lancet Psychiatry, the authors stated that in a group of 1052 psychiatrists surveyed, as many as 86% do not know the names of, and do not use, basic pharmacological databases such as Medscape or Epocrates in their daily work. This may be one of the most important factors causing the repeated hospitalisation of the examined persons or hindering the therapeutic effect in the group of psychiatric patients [20].

There is a lack of literature on the efficacy of using pharmacological interaction databases in psychiatry. Because of that, the main goal of this work was to investigate the effectiveness of the use of this type of database in psychiatrists from all over the world, i.e., from different continents. The second aim of this study was to make the studied group of psychiatrists aware of the role played by drug interaction databases in their daily work and, consequently, on the well-being of their psychiatric patients.

## 2. Methods

### 2.1. Studied Group and Procedure

The study involved 2146 psychiatrists from around the world. The psychiatrists were contacted mainly through the LinkedIn Recruiter website. Another way of conducting the survey was to contact psychiatric societies from different countries which then sent the developed survey to their members. Each psychiatrist was informed of the study aim before he or she agreed to participate. Participation in the study was voluntary.

The respondents completed a questionnaire that included questions about their sociodemographic data as well as those related to their daily medical practice. The survey also included 5 questions concerning case reports, which were answered by the respondents twice. The second time that the psychiatrists answered the same questions was after using the Medscape online database of drug interactions, where they could check whether or not there were dangerous drug interactions in the case reports. This made it possible to check the effectiveness of this database type in choosing the right answer, i.e., the one characterized by the lowest risk of a dangerous drug interaction in the presented case reports. These questions are included after the conclusion section of this report. The analysis only included questionnaires that included answers to all the questions. The selection of this database type was based on the fact that it is one of the most frequently used databases of this type, as well as the fact that it is publicly available [21,22]. The case reports were based on patients taking new psychoactive substances, one of the main problems faced by many countries around the world today. This choice was based on the fact that patients taking new psychoactive substances often combine them with other drugs, which unfortunately usually results in a series of different psychotropic drugs administered by psychiatrists to patients. Other questions concerned the use of pharmacological interaction databases, i.e., before and after the study. Under the literature, the text contains the 5 case reports analysed by psychiatrists.

### 2.2. Statistical Analysis

In order to check whether there are statistically significant differences in the percentage of correct answers given by the psychiatrists surveyed between the 2 time periods, the Wilcoxon test was used. When comparing 2 independent groups of people, the Mann–Whitney U test was used. The analysis using Kendall’s test made it possible to determine whether there is a statistically significant difference between the 3 aspects concerning the use and effectiveness of the pharmacological interaction database in the study group. The chi-squared test was used to check whether the groups of people being compared were equal.

## 3. Results

### 3.1. Characteristics of the Studied Group of Psychiatrists

Out of the 2146 psychiatrists, the largest part was made up of individuals from Europe, men, persons under 40 years of age, as well as those with 1–10 years of seniority (Table 1).

### 3.2. Effectiveness of the Pharmacological Interaction Database

**Figure 1:**
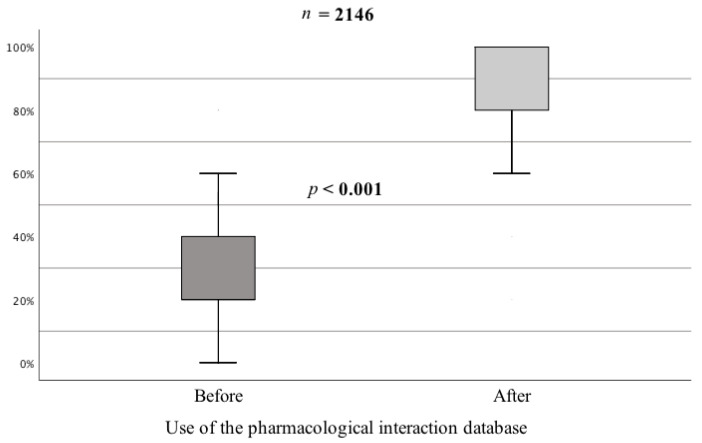
Percentage of correct answers given by psychiatrists to questions before and after using the pharmacological interaction database.

The mean level of correct answers given by the studied group of psychiatrists before using the pharmacological interaction database turned out to be statistically significantly lower compared to the period after using this database type, Z = 40.48; *p* < 0.001 (Figure 1).

Similar results apply to the analysis carried out on individual continents. It is important to note that the median percentage of correct answers given after using the pharmacological interaction database is 100 for all continents (Table 2).

Another noteworthy result shows that after using the pharmacological database, not a single person stated that they did not know the answers to specific questions. Before using this type of database, the majority of respondents indicated the wrong answer, while in the second part of the study, the majority indicated the correct answer (Table 3).

**Figure 2:**
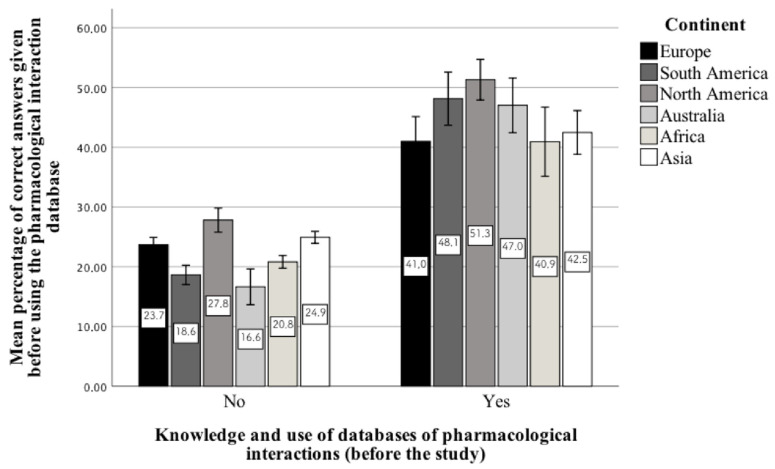
Mean percentage of correct answers given to the questions in the first stage of the study, i.e., in the group of psychiatrists who know and use the pharmacological interaction database on a daily basis or not.

In the group of psychiatrists from particular continents, i.e., those who know and use a database of pharmacological interactions, the mean percentage of correct answers given before using such a database turned out to be statistically significantly higher compared to psychiatrists who do not know or use them (Figure 2):

- Europe, U = 17,906; *p* < 0.001
- South America, U = 1448.5; *p* < 0.001
- North America, U = 3805.5; *p* < 0.001
- Australia, U = 104; *p* < 0.001
- Africa, U = 302; *p* < 0.001
- Asia, U = 5059; *p* < 0.001

### 3.3. Interest in the Pharmacological Interaction Database

It also turns out that after using the pharmacological interaction database, almost all the psychiatrists surveyed expressed interest in this database type and found that it is effective in optimizing the pharmacotherapy of psychiatric patients (Table 4).

Additionally, statistically significant differences were observed between groups of psychiatrists, categorized by their age and years of work experience. In the case of age, differences refer to the percentage of correct answers given before and after the use of the pharmacological interaction database. Psychiatrists between 61 and 80 years of age obtained a significantly lower (*p* < 0.001) percentage of correct answers before using the database in comparison with younger groups. After using the database, these differences were already significantly lower. Similar differences in the percentage of correct answers given before using the database apply to people working professionally the longest (*p* < 0.001). Compared to women, men achieved a higher percentage of answers, but this is only a minor difference (Table 5).

## 4. Discussion

The authors of the article published in Lancet indicate that over the last 25 years, there has been a growing burden put on the system related to mental diseases while at the same time not being the focus of the health sector in most countries [3]. In addition, the current COVID-19 pandemic shows negative effects on mental health, which unfortunately significantly increases the number of psychiatric patients. Among other things, there is a yearly increase in the number of hospitalisations of patients taking new psychoactive substances when combining them with other drugs, which very often results in the need for polypharmacotherapy [4,7]. The phenomenon of polypharmacotherapy increases the probability of subsequent patient hospitalisations, significantly affecting health results and health care resources. Since psychiatric patients also suffer from many other coexisting diseases, the increased use of psychotropic drugs creates an increased risk of clinically relevant interactions in these patients [23]. According to the information contained in the introduction, incorrectly conducted pharmacotherapy is an economic problem as removing its undesirable effects may turn out to be more expensive than proper treatment and often leads to rehospitalisation. Healthcare expenses are an increasingly difficult problem to solve, which affects all countries in the world. Hospitals generate enormous costs in the healthcare system. The financing of drugs is a large share of these costs [24,25]. Reducing these expenses can improve the financial condition of hospitals, and one way to achieve this is to use pharmacological interaction databases in daily practice. The very low percentage of psychiatrists using pharmacological interaction databases on a daily basis made us aware of the need to change this aspect. The studies we conducted are the first concerning the effectiveness of using a pharmacological interaction database in the field of psychiatry. In one of the most recent literature publications, i.e., published in JAMA, the authors recommend using a pharmacological interaction database as one of numerous majorly useful resources in the United States for the treatment of COVID-19 [26]. Our results indicate high effectiveness in using this type of database in the studied group of psychiatrists. So far, studies in psychiatry have included only single literature data in which the authors used a pharmacological interaction database to check the prevalence of possible side effects, namely those resulting from drug interactions. For example, in an article on Mexican patients with schizophrenia, the authors demonstrated the usefulness of a pharmacological interaction database for the simultaneous use of benzodiazepines and antidepressants [27]. In another study conducted on men and women over a period of 3 years in a psychiatric department, the authors used a pharmacological interaction database, i.e., Medscape, to illustrate the incidence of possible adverse reactions associated with drug interactions [28]. However, as previously mentioned, there are very few studies conducted by only a few authors working in the field of psychiatry. The high interest in the pharmacological interaction database after using it, and the opinion that it is effective in everyday work with psychiatric patients indicates the need to implement this type of tool in the medical environment. The increased awareness of the clinically relevant interactions of psychotropic drugs can help psychiatrists achieve optimal therapeutic outcomes for patients in primary health care settings. When conducting the study, it was also noted that a significant percentage of respondents did not know the answers to the questions concerning case reports. This may be due to the fact that prescribing is becoming more and more complex due to the increasing number of medications available, as well as information about their use, effectiveness, and side effects [29]. Although in recent years there has been significant progress made in teaching pharmacology, unfortunately, most medical universities do not provide students with the opportunity to practise prescribing medications in real life and do not consider them to be well-prepared for prescribing as young doctors. There are large differences in teaching pharmacology between the European Union member states [30]. Because of that, among other things, it seems necessary to have not only pharmacists but also the medical society, including psychiatric professionals, familiarised with the idea of everyday education concerning the use of pharmacological interaction databases.

## 5. Conclusions

Using the pharmacological interaction databases by psychiatrists could contribute to reducing the costs of treating psychiatric patients worldwide.

## 6. Limitations

**Figure 3:**
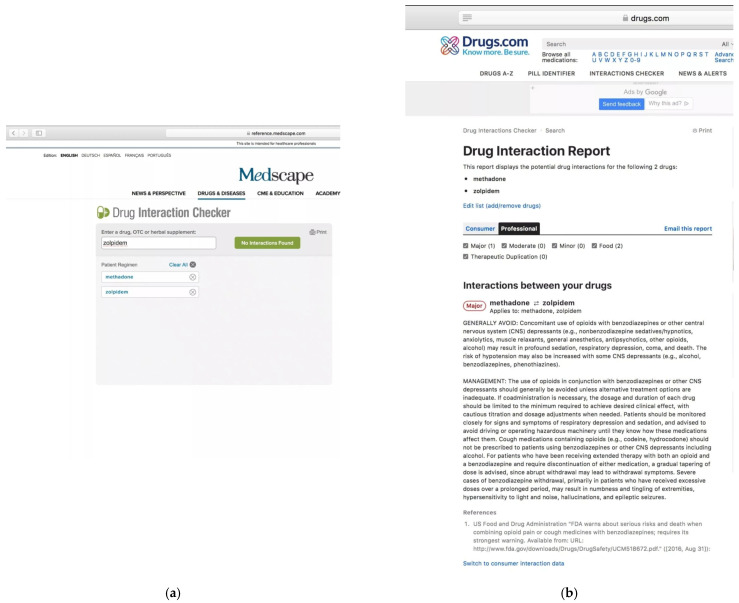
(**a**). Methadone–zolpidem interactions in the Medscape Drug Interaction Checker (last accessed on 8 December 2020), https://reference.medscape.com/drug-interactionchecker. (**b**). Methadone–zolpidem interactions in the Drug Interactions Checker (accessed on 4 May 2021), https://www.drugs.com/drug_interactions.html.

The limitation of this study is remote contact with participating psychiatrists. This is mainly due to the ongoing COVID-19 pandemic, and the resulting difficulties in performing stationary work, including participation in psychiatric conferences. Moreover, the databases of pharmacological interactions are not homogeneous and may contain discrepancies between additionally occurring drug interactions. However, due to the limited use of this type of databases by psychiatrists, the main goal was to encourage them to use this tool in their daily work in order to at least slightly increase their awareness of the possible side effects of drug interactions. To show the heterogeneity of drug interaction databases, and more specifically, the adverse effects resulting from drug interactions displayed in them, we give one example. It is related to the presented case report number one. According to the Medscape database, when entering methadone and zolpidem, there is no drug interaction between these drugs (Figure 3a), hence this drug was the correct answer. Another exemplary well-known drug interaction database is drugs.com (2021). When entering the same drugs, information about possible side effects resulting from their interactions appears (Figure 3b). Future research should focus on a thorough analysis and comparison of public drug interaction databases.

## 7. Case Reports

Due to the limitations of the Medscape pharmacological interaction database used in the manuscript, we compared the information it contained to the results obtained on drugs.com [31] and drugbank.com [32] databases. This allows for the visualisation of the heterogeneity of databases; however, as noted in this manuscript, the main goal was to demonstrate to psychiatrists from all over the world the aspect of using pharmacological interactions databases in their daily work.

1. A 22-year-old man has come to a primary care physician in a state of severe anxiety: he has run into the office, having jumped on some cars, shouting that he is being followed by agents. The patient has been participating in the methadone programme for several years (100 mg of methadone daily). The medicine that could be prescribed in this case is [21]:

Alprazolam (Increase in sedation)

Hydroxyzine (Increase in sedation and methadone toxicity through QTc interval)

Zolpidem

Lorazepam (Increase in sedation)

Oxazepam (Increase in sedation)

I do not know

Based on the Medscape pharmacological interaction database, zolpidem is the correct answer (Figure 3a), hence such an answer was considered correct here. However, additional information emerges when using other pharmacological interaction databases such as drugs.com and drugbank.com, i.e., the fact that simultaneous use of methadone with zolpidem may also cause deep sedation as well as respiratory failure.

2. A 35-year-old patient with paranoid schizophrenia has been re-admitted to a psychiatric hospital due to the ingestion of an unknown psychoactive substance, probably 3-MMC. He has been treated with olanzapine for several years. After admission to the psychiatric hospital, in order to reduce psychomotor agitation, the doctor should not prescribe [21]:

Clorazepate (Increase in sedation)

Oxazepam (Increase in sedation)

Temazepam (Increase in sedation)

Triazolam (Increase in sedation)

All the answers are correct

I do not know

According to the Medscape pharmacological interaction database, the simultaneous intake of olanzapine with the individual drugs listed as responses in the second case report may result in a drug–drug interaction adverse effect, i.e., in the form of increased sedation. Similar information on individual drug interactions is provided by the drugs.com and drugbank.com databases. However, these additional two databases show additional adverse effects which are decreased blood pressure, shallow breathing, muscle weakness, and slurred speech.

3. A 43-year-old patient has been admitted to a psychiatric hospital due to deterioration in his mental state caused by taking many new psychoactive substances, including traditional drugs, heroin, and amphetamine, for many days. The patient has difficulty falling asleep and his sleep is interrupted. For this reason, another doctor has prescribed zolpidem. During his previous visit to the hospital, he was diagnosed with an HCV infection. Therefore, the patient is taking ribavirin. Which of the following drugs should not be prescribed to the patient during his current hospitalization [21]?

Zaleplon

Eszopiclone 

Alprazolam (Pharmacodynamic synergy, additive CNS depression)

Hydroxyzine

Promazine

I do not know

In the case of the third case report, the Medscape database of pharmacological interactions indicates that a dangerous drug interaction may occur when zolpidem is taken concomitantly with alprazolam. However, two databases of pharmacological interactions, i.e., drugs.com and drugbank.com, indicate that the simultaneous use of zolpidem with zaleplon, eszopiclone, hydroxyzine, or promazine may cause dizziness, drowsiness, confusion, and difficulty concentrating (CNS depressant effect).

4. A 35-year-old patient was psychiatrically treated for many years for bipolar disorder and then for schizoaffective disorder. For 5 years, he was treated with clozapine (300 mg/day), and then for 6 months in combination with valproic acid (1000 mg/day). He also took mephedrone for several months. As a result, his mental condition worsened, and he was admitted to a psychiatric hospital. After examining the patient, a psychiatrist additionally prescribed hydroxyzine. Did he do the right thing [21]?

No, he did not, because hydroxyzine increases toxicity of clozapine by QTc interval

Yes, he did, because hydroxyzine can be taken simultaneously with the other listed drugs

No, he did not, because hydroxyzine and clozapine both increase sedation

Yes, he did, because the use of hydroxyzine reduces the risk of side effects resulting from the simultaneous intake of valproic acid and clozapine

The correct answers are the first and the third

I do not know

After entering the names of the two drugs, i.e., clozapine and hydroxyzine, into the Medscape pharmacological interaction database, two clinically significant interactions can be observed. They indicate that the correct answers in case number four are the first and the third. Making use of the remaining pharmacological interaction databases, the description of this type of interaction is further described. For example, the database of pharmacological interactions drugbank.com states that an increase in the QT and TdP intervals is observed only in patients with risk factors or in the case of intentional overdose. Factors that increase the risk of QTc prolongation include, but are not limited to, previous cardiovascular disease, low electrolyte levels, endocrine disorders, and kidney disease.

5. A 28-year-old patient has been suffering from deep depression for several weeks. She is taking mirtazapine. She has been admitted to a psychiatric hospital after taking a single dose of mephedrone. The medicine that could be prescribed in this case is [21]:

Oxazepam (Increase in sedation)

Flurazepam (Increase in sedation)

Promazine

Triazolam (Increase in sedation)

Hydroxyzine (Increase in sedation)

I do not know

In the case of the fifth case report, according to the Medscape database, the lack of pharmacological interaction concerns the simultaneous intake of mirtazapine with promazine. In the case of other drugs, the database indicates that sedation may be intensified. The information displayed by the other pharmacological interactions databases draws attention. An example here is the drugbank.com database informing that the simultaneous intake of mirtazapine with promazine may increase the risk of serotonin syndrome as well as hyperthermia. In the case of drugs.com, these are dizziness and difficulty concentrating, so the symptoms are also characteristic of serotonin syndrome.

## Figures and Tables

**Table 1:** Sociodemographic data of the examined persons.

Variable		*n*	%	Statistical Test Result *
Continent	Europe	771	35.9	χ^2^(5) = 849.91; *p* < 0.001
	South America	251	11.7	
	North America	398	18.5	
	Australia	109	5.1	
	Africa	145	6.8	
	Asia	472	22	
Sex	Male	1331	63.9	χ^2^(1) = 165.25; *p* < 0.001
	Female	775	36.1	
Age (years)	<40	1086	50.6	χ^2^(2) = 742.18; *p* < 0.001
	41–60	933	43.5	
	61–80	127	5.9	
Seniority (years)	1–10	1525	71.1	χ^2^(3) = 2576.27; *p* < 0.001
	11–20	427	19.9	
	21–30	155	7.2	
	>30	39	1.8	

* Chi-square test.

**Table 2:** Descriptive statistics on the percentage of correct responses given by the study group of psychiatrists from individual continents before and after using the pharmacological interaction database.

Continent	Percentage of Correct Answers Given: Before, after the Questionnaire	M (Mean)	SD (Standard Deviation)	Me (Median)	Statistical Test Result *
Europe	Before	25.94	17.87	20	Z = 24.36; *p* < 0.001
	After	90.69	14.41	100	
South America	Before	22.39	15.61	20	Z = 14.02; *p* < 0.001
	After	95.62	8.48	100	
North America	Before	31.45	19.86	40	Z = 17.42; *p* < 0.001
	After	86.93	12.86	80	
Australia	Before	22.2	17.92	20	Z = 9.13; *p* < 0.001
	After	93.39	9.44	100	
Africa	Before	23.86	10.35	20	Z = 10.65; *p* < 0.001
	After	93.93	9.22	100	
Asia	Before	27.33	12.49	20	Z = 19.12; *p* < 0.001
	After	87.2	14.59	100	

* Wilcoxon test.

**Table 3:** The studied group of psychiatrists providing a correct or incorrect answer to the questions asked or stating that they do not know the answers.

Use of the Pharmacological Interaction Database		Question									
		1		2		3		4		5	
		*n*	%	*n*	%	*n*	%	*n*	%	*n*	%
Before	Wrong answer	1510	70.4	1100	53.3	1132	52.7	879	41	1547	72.1
	Good answer	419	19.5	549	25.6	556	25.9	990	46.1	332	15.5
	I don’t know	217	10.1	497	23.2	458	21.3	277	12.9	267	12.4
Statistical test result *		χ^2^(2) = 1352.72; *p* < 0.001		χ^2^(2) = 312.17; *p* < 0.001		χ^2^(2) = 370.76; *p* < 0.001		χ^2^(2) = 411.51; *p* < 0.001		χ^2^(2) = 1453.33; *p* < 0.001	
After	Wrong answer	212	9.9	177	8.2	350	16.3	72	3.4	163	7.6
	Good answer	1934	90.1	1969	91.8	1796	83.7	2074	96.6	1983	92.4
	I don’t know	0	0	0	0	0	0	0	0	0	0
Statistical test result *		χ^2^(1) = 1381.77; *p* < 0.001		χ^2^(1) = 1496.39; *p* < 0.001		χ^2^(1) = 974.33; *p* < 0.001		χ^2^(1) = 1867.66; *p* < 0.001		χ^2^(1) = 1543.52; *p* < 0.001	

* Chi-square test.

**Table 4:** Psychiatrists’ knowledge of the pharmacological interaction databases before their use in the study, interest in them after the end of the study, and opinions that they can be effective in everyday work.

Continent	Knowledge and Use of Databases of Pharmacological Interactions (before the Study)		Interest in the Pharmacological Interaction Database (after Using it)		Efficacy of the Pharmacological Interaction Database in Daily Work (after Using It)		Statistical Test Result *
	*n*	%	*n*	%	*n*	%	
Europe	101	13.1	758	98.3	771	100	χ^2^(2) = 1269.44; *p* < 0.001
South America	32	12.7	248	98.8	251	100	χ^2^(2) = 432.08; *p* < 0.001
North America	62	15.6	391	98.2	398	100	χ^2^(2) = 656.34; *p* < 0.001
Australia	20	18.3	107	98.2	109	100	χ^2^(2) = 172.16; *p* < 0.001
Africa	22	15.2	144	94.3	145	100	χ^2^(2) = 242.05; *p* < 0.001
Asia	65	13.8	467	98.9	472	100	χ^2^(2) = 804.12; *p* < 0.001

* Kendall’s W test.

**Table 5:** Effect of age, professional service length, and sex on the percentage of correct answers given to the questions asked.

Variable		M (Mean)		SE (Standard Deviation)		Me (Median)		Statistical Test Result	
		Before	After	Before	After	Before	After	Before	After
Age (years) *	<40	25.1	89.2	20	100	16.09	14.05	χ^2^(2) = 804.12; *p* < 0.001	χ^2^(2) = 34.5; *p* < 0.001
	41–60	30.37	90.5	20	100	16.35	12.61		
	61–80	10.39	95.75	0	100	14.22	11.72		
Seniority (years) *	1–10	27.44	89.94	20	100	16.44	13.29	χ^2^(3) = 103.82; *p* < 0.001	χ^2^(3) = 6.29; *p* = 0.1
	11–20	28.43	90.68	20	100	16.04	13.37		
	21–30	16.13	90.16	20	100	17.07	13.93		
	>30	11.28	93.33	20	100	11.04	15.45		
Sex **	Male	27.73	91.4	20	100	17.91	12.72	U = 467,389; *p* < 0.001	U = 461,255; *p* < 0.001
	Female	24.38	87.95	20	100	14.35	14.26		

* Kruskal–Wallis test; ** Mann–Whitney U test.

## Data Availability

Please contact the corresponding author with any data availability requests.

## References

[1] Malhi G.S., Mann J.J. (2018). Depression. Lancet.

[2] OECD, European Union (2018). Promoting mental health in Europe: Why and how?. Health at a Glance: Europe 2018: State of Health in the EU Cycle.

[3] Patel V., Saxena S., Lund C., Thornicroft G., Baingana F., Bolton P., Chisholm D., Collins P.Y., Cooper J.L., Eaton J. (2018). The Lancet Commission on global mental health and sustainable development. Lancet.

[4] Peacock A., Bruno R., Gisev N., Degenhardt L., Hall W., Sedefov R., White J., Thomas K.V., Farrell M., Griffiths P. (2019). New psychoactive substances: Challenges for drug surveillance, control, and public health responses. Lancet.

[5] Schifano F., Orsolini L., Papanti G.D., Corkery J.M. (2015). Novel psychoactive substances of interest for psychiatry. World Psychiatry.

[6] Van Buskirk J., Griffiths P., Farrell M., Degenhardt L. (2017). Trends in new psychoactive substances from surface and “dark” net monitoring. Lancet Psychiatry.

[7] Ordak M., Nasierowski T., Muszynska E. (2019). The problem of poly-pharmacotherapy in patients on a mephedrone binge. Pharmacol. Res..

[8] Ordak M., Nasierowski T., Muszynska E., Bujalska-Zadrozny M. (2019). Optimisation of methadone treatment in a group of patients on a mephedrone binge and dependent on many psychoactive substances. Int. J. Psychiatry Clin. Pract..

[9] Maher R.L., Hanlon J., Hajjar E.R. (2014). Clinical consequences of polypharmacy in elderly. Expert Opin. Drug Saf..

[10] Scott I.A., Hilmer S.N., Reeve E., Potter K., Le Couteur D., Rigby D., Gnjidic D., Del Mar C.N., Roughead E.E., Page A. (2015). Reducing inappropriate polypharmacy: The process of deprescribing. JAMA Intern. Med..

[11] Hill-Taylor B., Walsh K.A., Stewart S., Hayden J., Byrne S., Sketris I.S. (2016). Effectiveness of the STOPP/START (Screening Tool of Older Persons’ potentially inappropriate Prescriptions/Screening Tool to Alert doctors to the Right Treatment) criteria: Systematic review and meta-analysis of randomized controlled studies. J. Clin. Pharm. Ther..

[12] Taylor S.R., Jones J.B., Shah N.R. (2008). Contracting for compliance: Using adherence as a patient-centered measure of performance. Am. Health Drug Benefits.

[13] Veeren J.C., Weiss M. (2016). Trends in emergency hospital admissions in England due to adverse drug reactions: 2008–2015. J. Pharm. Health Serv. Res..

[14] Hartholt K., Van Der Velde N., Looman C., Panneman M., Van Beeck E., Patka P., Van Der Cammen T. (2010). Adverse Drug Reactions Related Hospital Admissions in Persons Aged 60 Years and over, The Netherlands, 1981–2007: Less Rapid Increase, Different Drugs. PLoS ONE.

[15] Wawruch M., Zikavska M., Wsolova L., Kuzelova M., Tisonova J., Gajdosik J., Urbanek K., Kristova V. (2008). Polypharmacy in elderly hospitalized patients in Slovakia. Pharm. World Sci..

[16] Corsonello A., Pedone C., Corica F., Incalzi R.A. (2007). Polypharmacy in elderly patients at discharge from the acute care hospital. Ther. Clin. Risk Manag..

[17] Mizokami F., Koide Y., Noro T., Furuta K. (2012). Polypharmacy with Common Diseases in Hospitalized Elderly Patients. Am. J. Geriatr. Pharmacother..

[18] Wilfling D., Hinz A., Steinhäuser J. (2020). Big data analysis techniques to address polypharmacy in patients—A scoping review. BMC Fam. Pract..

[19] Błeszyńska E., Wierucki Ł., Zdrojewski T., Renke M. (2020). Pharmacological Interactions in the Elderly. Medicina.

[20] Ordak M., Nasierowski T. (2019). The pharmacological basis of drug interactions: An aspect overlooked in psychiatry. Lancet Psychiatry.

[21] (2020). Medscape: Drug Interaction Checker. https://reference.medscape.com/drug-interactionchecker.

[22] Day R.O., Snowden L. (2016). Where to find information about drugs. Aust. Prescr..

[23] Carmona-Huerta J., Obeso S.C.-D., Ramírez-Palomino J., Duran-Gutiérrez R., Cardona-Muller D., Grover-Paez F., Fernández-Dorantes P., Medina-Dávalos R. (2019). Polypharmacy in a hospitalized psychiatric population: Risk estimation and damage quantification. BMC Psychiatry.

[24] Morandi A., Bellelli G., Vasilevskis E.E., Turco R., Guerini F., Torpilliesi T., Speciale S., Emiliani V., Gentile S., Schnelle J. (2013). Predictors of Rehospitalization Among Elderly Patients Admitted to a Rehabilitation Hospital: The Role of Polypharmacy, Functional Status, and Length of Stay. J. Am. Med. Dir. Assoc..

[25] Sturmberg J.P., Bircher J. (2019). Better and fulfilling healthcare at lower costs: The need to manage health systems as complex adaptive systems. F1000Research.

[26] Sanders J.M., Monogue M.L., Jodlowski T.Z., Cutrell J.B. (2020). Pharmacologic Treatments for Coronavirus Disease 2019 (COVID-19). JAMA.

[27] Ocaña-Zurita M.C., Juárez-Rojop I.E., Genis A., Tovilla-Zárate C.A., González-Castro T.B., López-Narváez M.L., De La O De La O M.E., Nicolini H. (2016). Potential drug–drug interaction in Mexican patients with schizophrenia. Int. J. Psychiatry Clin. Pract..

[28] Patel T.K., Bhabhor P.H., Desai N., Shah S., Patel P.B., Vatsala E., Panigrahi S. (2015). Adverse drug reactions in a psychiatric department of tertiary care teaching hospital in India: Analysis of spontaneously reported cases. Asian J. Psychiatry.

[29] Gustafsson L.L., Wettermark B., Godman B., Andersén-Karlsson E., Bergman U., Hasselström J., Hensjö L.-O., Hjemdahl P., Jägre I., Julander M. (2011). The ‘Wise List’—A Comprehensive Concept to Select, Communicate and Achieve Adherence to Recommendations of Essential Drugs in Ambulatory Care in Stockholm. Basic Clin. Pharmacol. Toxicol..

[30] Brinkman D., Tichelaar J., Okorie M., Bissell L., Christiaens T., Likic R., Mačìulaitis R., Costa J., Sanz E., Tamba B. (2017). Pharmacology and Therapeutics Education in the European Union Needs Harmonization and Modernization: A Cross-sectional Survey Among 185 Medical Schools in 27 Countries. Clin. Pharmacol. Ther..

[31] Drugs.com: Drugs Interaction Checker 2021. https://www.drugs.com/drug_interactions.html.

[32] DrugBank: Drug Interaction Checker 2021. https://go.drugbank.com/drug-interaction-checker.

